# Investigation of the Antidiarrheal and Antimicrobial Activities of 80% Methanolic Leaf Extract of* Discopodium Penninervum* (Hochst.)

**DOI:** 10.1155/2018/1360486

**Published:** 2018-10-04

**Authors:** Dagninet Derebe, Mohammedbrhan Abdulwuhab, Muluken Wubetu, Faiz Mohammed

**Affiliations:** ^1^Department of Pharmacy, College of Medicine and Health Sciences, Bahir Dar University, Ethiopia; ^2^School of Pharmacy, College of Medicine and Health Sciences, Gondar University, Ethiopia; ^3^Department of Pharmacy, College of Health Sciences, Debre Markos University, Ethiopia; ^4^School of Medicine, College of Medicine and Health Science, Wolkite University, Wolkite, Ethiopia

## Abstract

Diarrhea is a major health problem throughout the world and it has become more problematic in developing countries like Ethiopia. People, in several parts of the world, use different traditional medicines for treating diarrhea and it has been reported that the roots, leaves, and flowers of various species are used for the same purpose. In Ethiopia, for instance,* Discopodium Penninervum* is used for the treatment of diarrhea and also to control infection. The aim of the present study was, therefore, to evaluate the in vivo antidiarrheal and in vitro antimicrobial effect of* Discopodium Penninervum* in mice. For the antimicrobial activity test, four standard bacteria and disc diffusion method were used, while for antidiarrheal experiment, animals had been used, which were divided into 5 groups. The first group served as negative control and was administered with vehicle (0.2-0.3ml of distilled water). Groups two (D100), three (D200), and four (D400) were administered 100, 200, and 400 mg/kg of the extract, respectively. Group five served as positive control group and was administered with either loperamide (3mg/kg) for castor oil induced diarrhea and castor oil induced enteropooling diarrhea models or atropine (1mg/kg) for charcoal meal test. Safety study was performed using a standard acute toxicity study procedure. The effect of the extract on castor oil induced diarrheal drops, onset of diarrhea, weight of faeces, small intestinal fluid accumulation, and intestinal motility was measured and analyzed using one-way ANOVA. Preliminary phytochemical screening of the leaves powder of the plant indicated the presence of various components. Inhibition of castor oil induced diarrhea was observed at all tested doses. It can be concluded that crude extracts of* Discopodium Penninervum* showed strong activities against diarrhea indicating that it contains some chemical constituents that possibly lead to antidiarrheal drug development.

## 1. Background

Diarrhea is the second most common cause of death in children under five years. It causes more than 5‐8 million deaths each year in infants and children below 5 years old [1]. Diarrhea is one of the most common causes of morbidity and mortality in many developing countries affecting mainly the infants and children's [2, 3].

Diarrhea is classified as acute or chronic, with acute diarrhea being the most common form. Infectious agents cause more than 90% of cases of acute diarrhea; these cases are often accompanied by vomiting, fever, and abdominal pain. The remaining 10% or so are caused by medications, toxic ingestions, ischemia, and other conditions. Viral* (adenovirus, enterovirus, and norovirus), *bacterial* (E. coli, V. cholerae, Shigella species, salmonella species, B. cereus, C. perfringens, Campylobacter species, S. aureus, and C. difficile), *and parasitic* (Cryptosporidium and Giardia) *agents are important pathogens, although* rotavirus* is the major cause of infectious diarrhea, particularly among young children [4–7].

**Figure 1:**
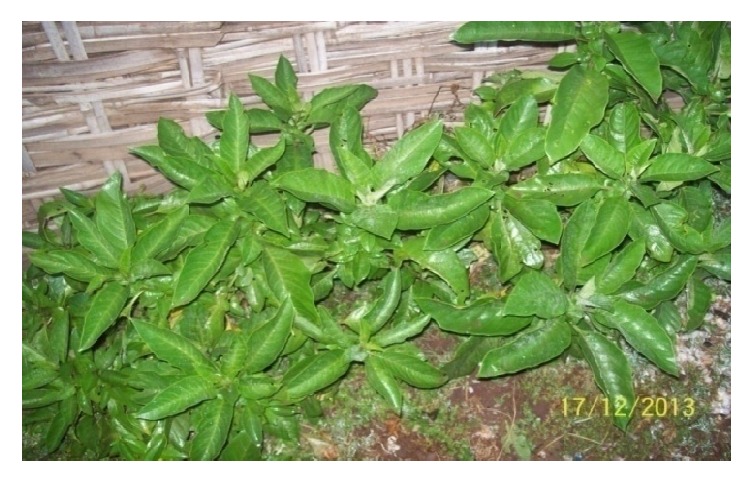
Plant of* Descopodium Penninervum* (Hochst.), Solanaceae.

Approximately one-half of all licensed drugs that were registered worldwide in the 25 years period prior to 2007 were natural products or their synthetic derivatives. Medicinal plants and their bioactive molecules are always in demand and are a central point of research. As a result, there is a recent surge in the demand for herbal medicine [8]. Meanwhile, a range of medicinal plants with antidiarrheal properties is widely used by traditional healers, but still there is an urgent need for research on medicinal plant in the management of diarrheal diseases.* Discopodium Penninervum* (Hochst.) (family: Solanaceae; local name: Aluma) is evergreen shrub (see Figure 1) found in many parts of Ethiopia. It is used for treatment of different types of livestock's and human diseases such as diarrhea, wound, snake bite, and liver disease [9–12]. Although a significant number of Ethiopians rely mainly on traditional medicines, generally, and* Discopodium Penninervum *leaves, particularly, for their primary healthcare needs against diarrhea, the effectiveness of these traditional medicines has not been scientifically evaluated [13–17]. Consequently, this study is intended to investigate the possible antidiarrheal (in vivo) and antimicrobial (in vitro) properties of the leaf extract of* Discopodium Penninervum *(Hochst.).

## 2. Materials and Methods

### 2.1. Plant Material

Fresh leaves of* Discopodium Penninervum *(100g) were collected during the month of December, from Dangila, 487 km North West of Addis Ababa, Ethiopia. The plant was identified by a taxonomist, and a voucher specimen (001) was deposited in the National Herbarium, College of Natural Sciences, Addis Ababa University.

Dried leaves of* Discopodium Penninervum *were thoroughly washed with distilled water to remove dirt, then dried under shade, and crushed into coarse powder. The powdered plant material was macerated in 80% of methanol for 72 h with occasional stirring. The filtrate was separated from the mark using Whatman Number 1 filter paper and the mark was remacerated three times. The filtrates were combined and dried in an oven at a temperature not exceeding 40°C. The dry extract was dark-brown and solid around 40°C. It was weighed by analytical balance and the yield was 16.67% (w/w). It was then transferred into vials and kept in desiccators until use [18, 19].

For this study, 80% of methanol, instead of water, was used to get greater percent of extract yield based on previous studies conducted. Importantly, methanol serves as a less medium for the occurrence of the microorganisms; it is more efficient in cell wall and seed degradation as well as having low or no enzyme activity as compared to water [20]. Additionally, alcoholic or hydroalcoholic extract of plant materials contain a wide variety of polar (and moderately polar) compounds [21, 22].

#### 2.1.1. Preliminary Phytochemical Screening

Standard tests were employed to detect the major secondary metabolites such as phenolic compounds, saponins, flavonoids, tannins, sterols, resins, alkaloids, cardiac glycosides, and terpenoids [20, 23].

### 2.2. Experimental Animals

A total of 95 adult albino mice of both sexes were used for the whole experiment and they were obtained from colonies in the animal unit of the University of Gondar, College of Medicine and Health Sciences, Department of Pharmacology. They were 6-8 weeks of age and had weights ranging from 20 to 36 g. They were housed in plastic cages with standard wood chip bedding and had access to food and water* ad libitum*. Light was in its natural cycle (for a 12 h on and 12 h off). Care and handling of the mice were performed according to the guidelines given by OECD and ILAR [24–26].

### 2.3. Acute Toxicity Test

The crude extract was evaluated for its toxicity in young female, nulliparous, and nonpregnant Swiss albino mice aged 6-8 weeks and weighing 28-34g. Oral Acute toxicity study was conducted based on the limit test as per the internationally accepted protocol drawn by OECD [27]. The mice were kept for one week prior to dosing to acclimatize and then fasted for 3 hours food but with normal supply of water. The fasted body weight (BW) of each animal was determined and the doses were calculated according to the body weight. The test substance was administered at 2000mg/kg by oral route. After the administration, food was withheld for a further 2 hours. Animals were observed continuously for physical or behavioural changes during the first 30 min after dosing and observed periodically (with special attention given during the first 4 hours) for the next 24 hours and then daily thereafter for 14 days. Following the results from the first mouse, other two mice were recruited, fasted for 3 h, administered a single dose of 2000 mg/kg, and observed in the same manner. These observations continued for further 14 days for any signs of overt toxicity, and individual weights of animals were recorded before the administration of drug on 1st day of the study and thereafter on the 7th and 14th day of the experiment [27].

### 2.4. Antimicrobial Assay

Four pathogenic bacteria, namely,* Shigella species ATCC 9289, E.coli* ATCC 1912/R,* Salmonella typhi ATCC25922*, and* Staphylococcus aureus *ATCC259223 were used to test organisms for antibacterial activity of the dried extract. The bacterial strains were obtained from the Microbiology Laboratory of Gondar University. Nutrient agar media was used for culture of the test organisms, the antibacterial activity was determined by disc diffusion method, and plant extract of 200*μ*g/ml was used for comparison with standard ciprofloxacin discs of 5*μ*g/disc. At the end of incubation, inhibition zones formed around the disc were measured with transparent ruler in millimeter. These studies were performed in triplicate and the average diameter of zone of inhibition was obtained.

### 2.5. Antidiarrheal Activity

#### 2.5.1. Animal Grouping and Dosing

In all models, the animals were randomly divided into five groups of six mice in each group. The first group served as negative control and was administered with vehicle (0.2-0.3ml of distilled water based on their weight). Groups two (D100), three (D200), and four (D400) were administered 100, 200, and 400 mg/kg of* Discopodium Penninervum *extract, respectively. Group five served as positive control group and was administered with either loperamide (3mg/kg) for castor oil induced diarrhea and castor oil induced enteropooling diarrhea models or atropine (1mg/kg) for charcoal meal test. The administration for all groups was done orally by using oral gages.

### 2.6. Pharmacological Screening

#### 2.6.1. Castor Oil Induced Diarrhea

A total of thirty mice were fasted for 12 hours before commencing the experiment with free access to water. The mice were randomly divided into five groups, six in each group. Group I served as a negative control, while D100, D200, and D400 were administered 100 mg/kg, 200 mg/kg, and 400 mg/kg serially double doses of the methanolic extract of* Discopodium Penninervum,* respectively, and Group five were administered suspension form of loperamide (3 mg/kg). Then the animals were kept in separate cages with a transparent plastic container beneath the cage to collect faeces.

The watery fecal material, onset of defecation, and frequency of defecation were noted up to 4 hours in the transparent metabolic cages with preweighed plastic dishes placed at the base. Weight of plastic dish before and after defecation and total number of faeces expelled were noted and compared to control.

### 2.7. Castor Oil Induced Enteropooling

A total of thirty mice were randomly selected, fasted for 18 hours before the commencement of the experiment, and randomly allocated into five groups of six mice in each group. Group I served as a negative control, while D100, D200, and D400 were administered 100 mg/kg, 200 mg/kg, and 400 mg/kg serially double doses of the methanolic extract of* Discopodium Penninervum,* respectively, and Group five was administered suspension form of loperamide (3 mg/kg). After 30 minutes of drug treatment, the animals of each group received 0.3ml of castor oil orally. Then 30 minutes later, the mice were sacrificed by cervical dislocation; the whole length of small intestine from pylorus to caecum was removed immediately and intestinal fluid was collected and weighed.

### 2.8. Small Intestinal Transit

The effect of the extract on small intestinal transit was studied in thirty overnight fasted mice which were divided randomly into five different groups, six mice in each group. Group I served as a negative control, while D100, D200, and D400 were administered 100 mg/kg, 200 mg/kg, and 400 mg/kg serially double doses of the methanolic extract of* Discopodium Penninervum,* respectively, and Group five was administered Atropine Sulphate (1 mg/kg, orally), 1 hour before the administration of castor oil. Thirty minutes after the treatment, each of these animals was given 0.3 ml of charcoal meal (3% charcoal suspension in 5% suspension of acacia) by oral route. Then each animal was sacrificed 30 minutes later and the abdomen was opened. The percentage distance of the small intestine (from the pylorus to the caecum) traveled by the charcoal plug was determined and the distance traveled by charcoal with reference to total length was calculated to express the percentage of distance traveled. The percentage inhibition of intestinal transit by the extract was calculated.

### 2.9. Statistical Analysis

Data obtained from the study are presented as mean ± standard error of mean. Data analysis was performed using Statistical Package for Social Science (SPSS), version 20. Comparison of mean of total fecal output, mean delay in onset of diarrhea, mean number of defecation, mean of weigh of gastric contents, and mean of distances traveled by charcoal meal longitudinally along the small intestine among the control and extract treated groups were determined by one-way ANOVA followed by post hoc Tukey method. The significance was set at p<0.05. Then the percentage of diarrheal inhibition, percentage inhibition of weight of intestinal contents, and percent inhibition of intestinal motility were also calculated for comparison purpose.

## 3. Results

### 3.1. Phytochemical Study

The result of preliminary phytochemical screening of the methanolic leaf extract of* Discopodium Penninervum *is presented in Table 1 and it revealed that the extract contains flavonoids, terpenoids, phenols, saponins, and tannins, whereas cardiac glycosides, sterols, and alkaloids were absent.

### 3.2. Acute Toxicity Study

Acute toxicity study of the hydroalcoholic extract of* Discopodium Penninervum *was conducted through oral administration of a single dose of 2000 mg/kg. Gross physical and behavioural observation of the experimental mice revealed no visible signs of behavioural, neurological, autonomic, or physical changes. Besides, the extract did not cause mortality. Thus, the median lethal dose (LD50) of the plant extract can be said to be greater than 2000 mg/kg, indicating a good safety margin.

### 3.3. Antimicrobial Activity

Antibacterial activity of extract of* Discopodium Penninervum *leaves was studied on one Gram positive and three Gram negative bacteria by disc diffusion method and compared with the standard antibiotic disc. Antibacterial activity of* Discopodium Penninervum *leaves extract was measured at 200 *μ*g/ml concentration. The extract exhibited high activity against* Shigella species ATCC 9289* and poor activity against* E.coli ATCC 1912/R*, but no activity against* Staphylococcus aureus ATCC259223* and* Salmonella typhi ATCC25922.* More specifically,* Discopodium Penninervum *leaves extract showed 20 and 7 mm diameter of zone of inhibition against* Shigella species ATCC9289 *and* E.coli ATCC1912/R*, respectively, and no activity against* Staphylococcus aureus ATCC259223 *and* Salmonella typhi ATCC25922 *tested. Standard antibiotic ciprofloxacin (5 *μ*g/disc) showed significant antibacterial activity against all Gram positive and Gram negative bacteria tested (Table 2).

### 3.4. Castor Oil Induced Diarrhea

**Figure 2:**
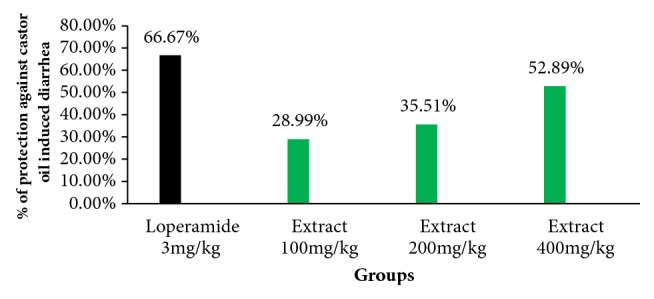
Percentage protection against castor oil induced diarrhea by methanolic extracts of leaves of* Discopodium Penninervum.*

The antidiarrheal results of hydroalcoholic extract of the leaves of* Discopodium Penninervum *in Swiss albino mice are shown in Table 3 and Figure 2. When compared with the respective negative control group (Group I), the oral doses of the extract D100, D200, and D400 prolonged the onset time of diarrhea significantly (P<0.05) (i.e., delayed the onset time of defecation) (52.00±2.83, 58.17±2.04, and 84.67±7.61 minutes, respectively). The delay in the onset of diarrhea was p<0.01 in D200 and D400 and p<0.05 in D100.

The extract also decreased the number of defecation at all tested dose as shown in Table 3. At a dose of 100, 200, and 400mg/kg, the extract significantly decreased (p<0.05) the total number of faeces produced upon administration of castor oil (7.83±1.47, 6.67±1.63 and 5.17±1.17, respectively) compared to the control group (23.83±8.52). The weight of defecation for D100, D200, and D400 was 0.98±0.06, 0.89±0.06, and 0.65±0.21, respectively, and was reduced significantly (p<0.05) when compared with Group I (1.38±0.33) as stated in Table 3.

The percent of diarrheal inhibition of the extract was 28.99%, 35.51%, and 52.89% at a dose of 100mg/kg, 200mg/kg, and 400mg/kg, respectively, at the serial dose of the extract (Figure 2). The percent of inhibition of the loperamide was 66.67% which was more than the large dose of the extract.

### 3.5. Castor Oil Induced Enteropooling

**Figure 3:**
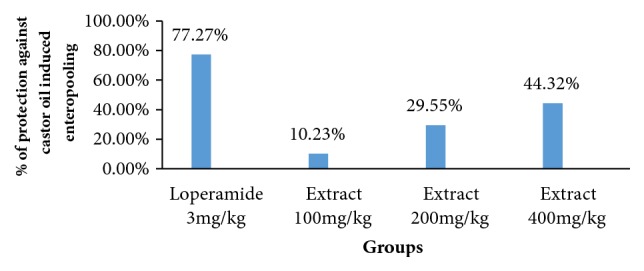
Percentage protection against castor oil induced enteropooling by methanolic extracts of leaves of* Descopodium Penninervum.*

The extract significantly (p<0.01) inhibited castor oil induced diarrhea at a dose of 200 mg/kg and 400 mg/kg in mice as shown in Table 4 and Figure 3. Weight of intestinal fluid in Group I was 0.88±0.08 and was 0.8±0.07, 0.62±0.05, and 0.49±0.06 in D100, D200, and D400, respectively (Table 4).

The percentage reduction of enteropooling exhibited by D100, D200, and D400 of extract was 10.23%, 29.55%, and 44.32%, respectively, whereas the loperamide reduces volume accumulation by 77.27% (Figure 3). The reduction of intestinal volume content by the extract was very significant (p<0.01) at the dose of 200mg/kg and 400mg/kg.

### 3.6. Gastrointestinal Motility Test

**Figure 4:**
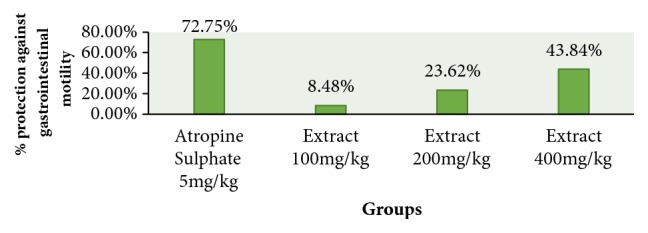
Percentage protection against gastrointestinal motility by methanolic extracts of leaves of* Discopodium Penninervum.*

The methanolic leave extract of* Discopodium Penninervum *significantly decreased the propulsion of charcoal meal through the gastrointestinal tract when compared with control as shown in Table 5 and Figure 4. The percentage of charcoal movement from pylorus to caecum was 78.77±10.64, 65.74±8.92, and 48.34±6.70 in D100, D200, and D400, respectively.

Percentage inhibition of charcoal movement in GIT by 100 mg/kg, 200 mg/kg, and 400 mg/kg of the methanolic extract was 8.48%, 23.62%, and 43.84%, respectively, whereas Atropine Sulphate inhibits charcoal transit by 72.75%. At the dose of 200mg/kg and 400mg/kg the percentage of inhibition movement of charcoal from pylorus to caecum was very significant (p<0.01).

## 4. Discussion

The present study sought to assess the antidiarrheal and antimicrobial activity of the plant. Typical acute toxicity (such as changes in general behaviours, variations in body weight, and mortality) with the test extract was not observed during the given period of test [28]. Since the maximum dose level recommended by OECD is 2000 mg/kg body weight, further dosing to estimate the median lethal dose (LD_50_) of the plant was not performed. Actually, at the end of 14 days no death and no adverse effects of extract were recorded at 2000mg/kg, which was about 5 times the maximum effective dose tested (400 mg/kg). Based on the result, it can be stated that* Discopodium Penninervum's* LD50 value is greater than 2000mg/kg and this plant can be classified under category 5 in accordance with Globally Harmonised System of Classification and Labelling of chemicals (GHSCL) [29].

The results of the study showed that the extract of* Discopodium Penninervum *produced a statistically significant reduction in the frequency of diarrhea produced by castor oil. The extract significantly reduced the intestinal transit significantly as revealed by decrease in the portion of intestinal length traversed by charcoal meal. It is also noted that the methanolic extract of the* Discopodium Penninervum *leave had activity against diarrhea causing bacteria such as* Shigella species ATCC 9289* and poor antimicrobial activity against* E.coli ATCC 1912/R*.

Although the extract was found to reduce castor oil induced diarrheal episodes, enteropooling, and intestinal motility, mechanism of its activity is uncertain but we can speculate based on mechanism of castor oil induced diarrhea. Diarrhea induced by castor oil occurs when this castor oil hydrolysis into ricinoleic acid is catalyzed by intestinal lipases. Subsequently, the antidiarrheal effect of the plant extract may be by inhibition of ricinoleic acid production and/or antagonizing the action of ricinoleic acid or inhibiting the production of prostaglandin or antagonizing the action of prostaglandin. The* Discopodium Penninervum *might be enhancing the absorption of water, electrolytes, and glucose and also brings the antisecretory effect of intestinal mucosa as a result of this antidiarrheal effect [30, 31]. The plant extracts have also ability to inhibit the peristaltic movement of gastrointestinal tract, resulting in the antimotility effect which in turn allows the absorption of water and electrolytes as a result of this antidiarrheal effect. This effect of the extracts might be due to inhibition of acetylcholine effect on gut.

Loperamide is used as the standard drug in this study as it stimulates absorption of fluid, electrolytes, and glucose and reversed the effect of prostaglandin. Another standard drug atropine significantly reduces intestinal transit time due to its anticholinergic effect. However, it does not inhibit castor oil induced enteropooling, thereby suggesting that mediators other than acetylcholine are involved in castor oil induced enteropooling. Furthermore, a decrease in intestinal transit time with atropine could also be due to reduction in gastric emptying [32, 33].

Although mechanism of action of these secondary metabolites was not evaluated in the present study, its antidiarrheal activity might be attributed to the presence of phytochemical constituents such as flavonoids, terpenoids, phenols, saponins, and tannins. Tannins, flavonoids, phenols, and saponins in general have been reported to have antidiarrheal activity from other similar studies, whereas tannins, flavonoids, and phenols have been reported to have antimicrobial activity [20, 34]. The antidiarrheal activity of flavonoids has been ascribed to their ability to inhibit release of autacoids and prostaglandins. The antimicrobials activity of flavonoids was due to the ability to form complex with cell wall, binding to adhesion [35, 36].

The antidiarrheal activity of tannins has been ascribed to their ability to make intestinal mucosa more resistant, reduce secretion, and stimulate normalization of deranged water transport across the mucosal cells and reduction of the intestinal transit. The antimicrobial activity was due to their ability to bind to adhesins, enzyme inhibition, substrate deprivation, complex with cell wall, membrane disruption, and metal ion complexation [20].

The antidiarrheal activity of terpenoids is because of their ability to inhibit the release of autacoids and prostaglandins. Terpenoids have also antimicrobial activity by membrane disruption. Saponins inhibit histamine release in vitro by which it exhibits antidiarrheal activity [20].

## 5. Conclusion

This study collectively indicates that the hydroalcoholic extract of* Discopodium Penninervum* is not toxic and does not cause major acute toxic symptoms on acute toxicity test bases. The extract also possesses promising antidiarrheal activity and has high antimicrobial activity against* Shigella species ATCC 9289* and poor antimicrobial activity against* E.coli* ATCC 1912/R, which can be more evaluated for its profound activities by further researches. The plant contains flavonoids, terpenoids, phenols, saponins, and tannins, but alkaloids, sterols, and cardiac glycosides are absent.

## Figures and Tables

**Table 1:** Results of phytochemical screening of methanolic leaf extract of *Discopodium Penninervum.*

S.no	Tests	extract
1	Flavonoids	+

2	Terpenoids	+

3	Phenols	+

4	Saponins	+

5	Tannins	+

6	Cardiac glycosides	_

7	Sterols	_

8	Alkaloids	_

(+) present, (-) absent.

**Table 2:** Antimicrobial activity of *Discopodium Penninervum extract *against four standard bacteria.

Names of the bacteria	Methanol extract 200 *μ*g/ml. (Zone of inhibition) mm in diameter	Ciprofloxacin 5 *μ*g/disc (Zone of inhibition) mm in diameter
*Shigella species ATCC 9289*	20	22

*E.coli ATCC 1912/R*	7	27

*Salmonella typhi ATCC25922*	0	21

*Staphylococcus aureus* ATCC259223	0	11

**Table 3:** Evaluation of antidiarrheal activity of methanolic extracts of leaves of *Discopodium Penninervum *against castor oil induced diarrhea.

Gp no.	Dose	Onset of diarrhea	Total number of faeces	Weight of fresh fecal output
Group_1(-ve control)_	-	23.83±8.52	11.67±2.80	1.38±0.3
Group_2(D100)_	100mg/kg	52.00±2.83*∗*	7.83±1.47*∗*	0.98±0.06*∗*
Group_3(D200)_	200mg/kg	58.17±2.04*∗∗*	6.67±1.63*∗∗*	0.89±0.06*∗∗*
Group_4(D400)_	400mg/kg	84.67±7.61*∗∗*	5.17±1.17*∗∗*	0.65±0.21*∗∗*
Group_5(+ve control)_	3mg/kg	138±33.75*∗∗*	3.00±1.79*∗∗*	0.46±0.32*∗∗*

Key: data are expressed as means ± SEM for six mice per a group; *∗∗* means p<0.01; *∗* means p<0.05.

**Table 4:** Evaluation of anti-diarrheal activity of methanolic extracts of leaves of *Discopodium Penninervum *against castor oil induced enteropooling.

Group numbers	Dose (mg/kg)	Weight of intestinal fluid (ml)
Group_1(-ve control)_	-	0.88±0.08
Group_2(D100)_	100mg/kg	0.79±0.07^ns^
Group_3(D200)_	200mg/kg	0.62±0.05*∗∗*
Group_4(D400)_	400mg/kg	0.49±0.06*∗∗*
Group_5(+ve control)_	3mg/kg	0.20±0.08*∗∗*

Key: data are expressed as means ± SEM for six mice per a group; *∗∗* means p<0.01; *∗* means p<0.05; ns: p>0.05, not significant.

**Table 5:** Evaluation of antidiarrheal activity of methanolic extracts of leaves of *Descopodium Penninervum *in gastrointestinal motility test.

Groups	Dose	% Traversed by charcoal meal
Group_1(-ve control)_	-	86.07±8.59
Group_2(D100)_	100mg/kg	78.77±10.64^ns^
Group_3(D200)_	200mg/kg	65.74±8.92*∗∗*
Group_4(D400)_	400mg/kg	48.34±6.70*∗∗*
Group_5(+ve control)_	3mg/kg	23.45±8.37*∗∗*

Key: data are expressed as means ± SEM for six mice per a group; *∗∗* means p<0.01; *∗* means p<0.05; ns: p>0.05, not significant.
