# Corrigendum to “Investigation of the Antidiarrheal and Antimicrobial Activities of 80% Methanolic Leaf Extract of* Discopodium penninervum* (Hochst.)”

**DOI:** 10.1155/2018/4915802

**Published:** 2018-11-01

**Authors:** Dagninet Derebe, Mohammedbrhan Abdulwuhab, Muluken Wubetu, Faiz Mohammed

**Affiliations:** ^1^Department of Pharmacy, College of Medicine and Health Sciences, Bahir Dar University, Ethiopia; ^2^School of Pharmacy, College of Medicine and Health Sciences, Gondar University, Ethiopia; ^3^Department of Pharmacy, College of Health Sciences, Debre Markos University, Ethiopia; ^4^School of Medicine, College of Medicine and Health Science, Wolkite University, Wolkite, Ethiopia

In the article titled “Investigation of the Antidiarrheal and Antimicrobial activities of 80% Methanolic leaf extract of* Discopodium penninervum* (Hochst.)” [1], Dr. Faiz Mohammed was missing from the authors' list. He helped in preparing the literature reviews. He also contributed to the writing and editing of the article, commenting on it and approving its integrity and accuracy. The corrected authors' list is shown above and corrected in place.

Additionally, the following Acknowledgments are added to the article:

The authors would like to thank Dr. Nurhamed Mohammed for his role in the laboratory work.
